# An Atypical Presentation of Extragenital Lichen Sclerosus et Atrophicus

**DOI:** 10.5826/dpc.1104a72

**Published:** 2021-10-01

**Authors:** Sabina Vaccari, Alessia Barisani, Francesca Pepe, Carlotta Baraldi, Bianca Maria Piraccini, Valeria Gaspari

**Affiliations:** 1 Dermatology, IRCCS Policlinico di Sant’Orsola, Department of Experimental, Diagnostic and Specialty Medicine, Alma Mater Studiorum Università di Bologna, Italy

**Keywords:** extragenital lichen sclerosus et atrophicus, morphea, dermoscopy, histopathology

## Introduction

Lichen Sclerosus et Atrophicus (LSA) is a chronic inflammatory, invalidating disorder mainly involving the anogenital skin. Extragenital lesions are uncommon, occurring as multiple, oval, porcelain-white macules, or papules. When present, they are mostly observed on the superior trunk, axillae, buttocks, and extremities, following Blaschko lines [1]. In the literature, there is a high prevalence of coexistence of LSA and morphea [2].

## Case Presentation

**Figure 1:**
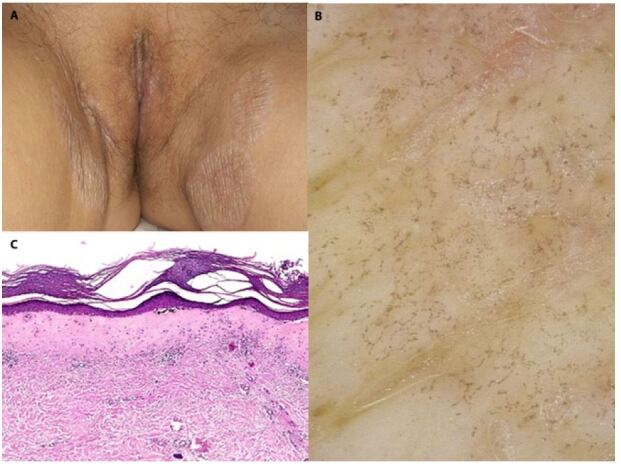
(A) Extragenital lichen sclerosus et atrophicus with porcelain-white color plaques. (B) Dermoscopic patterns: abundant peppering over white-yellowish structureless areas (magnification × 20). (C) Histological examination: thick hyperkerator scale an atrophic epidermis and a pale superficial dermal stroma with rare lymphocytes and plasma cells; melanophages displaced in upper dermis.

**Figure 2:**
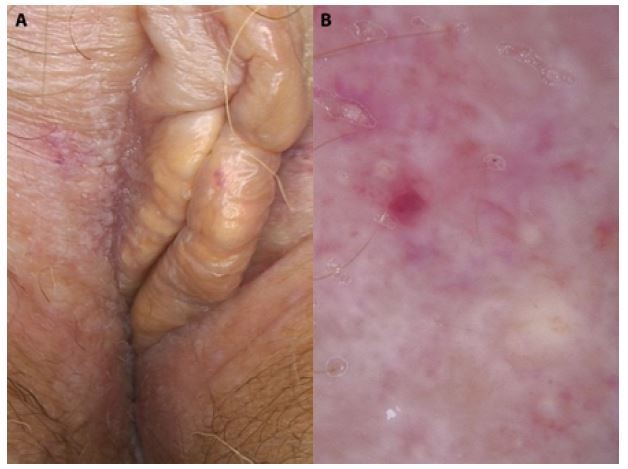
(A) Genital lichen sclerosus et atrophicus with patches of sclerosis and purpuric lesions. (B) Dermoscopic patterns: red purpuric blotches corresponding to ecchymosis in an intensely white background.

We report the case of a 70-year-old woman who presented bilateral symmetrical itchy lesions affecting the inner thighs, appearing as wrinkled, porcelain-white color plaques with cigarette-paper-like texture and measuring 4 cm × 4 cm (Figure 1A). The lesions had been present for about 1 year. Moreover, an involvement of the vulvar mucosa, with loss of clitoris and fusion between the labia majora and minora, was observed, as it happens in genital lichen sclerosus et atrophicus (LSA) (Figure 2A).

Dermoscopy of cutaneous lesions revealed widespread whitish patches, comedo-like openings, and peppering patterns (Figure 1B). On dermoscopy, the vulvar lesions showed patchy structureless milky-pinkish areas with scales and red purpuric blotches.

The differential diagnosis of the skin patches included morphea and extragenital LSA, due to their clinical morphology and distribution and to the association with anogenital lesions [3]. A punch biopsy of cutaneous lesions showed a thick hyperkeratotic scale and an atrophic epidermis with flattening of the rete ridges. The basal layer cells showed hydropic degeneration. Beneath the epidermis there was a broad area of pronounced lymphedema. Within this zone, the collagenous fibers were swollen and homogenous and contained only a few nuclei. Dermal melanophages, rare lymphocytes, and plasma cells were observed (Figure 1C). Based on the clinical and histological findings, a final diagnosis of extragenital LSA was made. A vulvar biopsy was also performed, confirming the diagnosis of genital LSA (Figure 2B).

## Conclusions

Extragenital LSA affects about 15–20% of patients with genital LSA. It is generally asymptomatic and usually affects neck, shoulders, and upper trunk, presenting as flat, white papules or slight atrophic white plaques. The diagnosis is mainly clinical and is confirmed by histopathology.

Clinically, our patient showed a specific skin lesions that made us hypothesize an inflammatory disease, such as morphea or extragenital LSA, but due to symmetrical distribution and the peculiar size, the lesions were not pathognomonic for any disease.

The uncertainty in diagnosing extragenital LSA was due to the uncommon sites of the lesions, very close to anogenital LSA, rather than on the trunk or sites of pressure. On the contrary, dermoscopy added features suggesting the hypothesis of extragenital LSA, showing well-demarcated, whitish structureless areas, and comedo-like openings [4]. Dermoscopy also allowed the differential diagnosis with morphea since the latter usually shows fibrotic beams and linear branching vessels on dermoscopy (5). A peculiar dermoscopic feature in our case was the peppered arrangement, which is usually observed only in vulvar LSA [6]: it is thought to be related to melanophages displaced in both the upper dermis and perifollicular site. Thus, peppering may represent an additional dermoscopic clue in the differential diagnosis between morphea and extragenital LSA.

Further cases of extragenital LSA need to be reported and collected, to deepen the understanding of this rare disease’s dermoscopic features, confirming the presence of peppering as clue for differential diagnosis.
